# Research on the optimization of cold chain logistics distribution routes considering time-dependent networks and simultaneous pick-up and delivery from the perspective of sustainability

**DOI:** 10.1371/journal.pone.0330535

**Published:** 2025-09-05

**Authors:** Yanqiu Liu, Jiaqi Hou, Chao Cai

**Affiliations:** School of Management, Shenyang University of Technology, Shenyang, Liaoning, China; National Taiwan University of Science and Technology, TAIWAN

## Abstract

In today’s economic globalization, the cold chain logistics industry is a fundamental sector supporting the development of the national economy, with sustainable development and a people-oriented approach being crucial. This paper investigates the path optimization problem of cold chain logistics vehicles that simultaneously pick up and deliver goods within a time window, aiming to achieve sustainable development in real-world cold chain logistics distribution. Additionally, the paper takes into account the varying speeds of vehicles over time to more accurately simulate real-world traffic conditions. The objective of the mathematical model is to minimize the total cost, which includes economic, environmental, social, and equity costs. To address this problem, a Heuristic Cross Brainstorm Optimization Algorithm (HCBSO) has been selected, and strategies such as segment-based initial solution construction and heuristic crossover have been designed to enhance the algorithm’s performance. Furthermore, a departure time optimization strategy has been developed to avoid traffic congestion. The effectiveness of the model and algorithm has been verified through multiple sets of comparative tests. The test results indicate that the constructed model and designed algorithm can scientifically optimize departure times, plan vehicle routes to avoid traffic congestion, effectively reduce total costs, and promote the sustainable development of the cold chain logistics system.

## 1 Introduction

Cold chain logistics plays a vital role in safeguarding the quality and safety of temperature-sensitive products such as fresh produce and pharmaceuticals. However, the sector faces multifaceted challenges, including high operational costs, contamination risks, and heightened time sensitivity. As consumer expectations for product freshness and on-time delivery continue to rise, return rates have also increased, placing greater demands on the responsiveness and adaptability of cold chain systems. Among these challenges, strict adherence to time-window constraints has emerged as a critical factor, as even a one-hour delay in delivery can increase spoilage rates by up to 15%. This issue is particularly pronounced in urban areas, where growing vehicle ownership has exacerbated traffic congestion, resulting in significant temporal variability in road conditions. In such a dynamic environment, vehicle routing in cold chain logistics must be capable of real-time adjustment to traffic fluctuations in order to mitigate delays and maintain product integrity. Beyond these operational concerns, cold chain optimization is increasingly recognized as essential to global sustainability. Notably, cold chain transportation accounts for over 40% of total logistics energy consumption, primarily due to the widespread use of diesel-powered refrigerated trucks, which not only inflate operating costs but also significantly contribute to carbon emissions. In recent years, green logistics has become a prominent research focus, aiming to achieve cost reduction, efficiency improvement, and waste minimization from the perspective of logistics companies. The highly polluting nature of cold chain logistics has also attracted significant attention from scholars. For example, Leng et al. aimed to address the dual-objective low-carbon siting-path problem of cold chain logistics. An optimization model of the comprehensive cold chain low-carbon siting-path problem was established, and multi-objective heuristics were designed for solving it [1]. Wang et al. addressed the challenges of precise monitoring and low-carbon sustainable routing in fruit cold chain logistics, proposing a flexible temperature-sensing solution that is integrated with an intelligent cold chain control strategy, aiming to achieve effective cold chain food quality management and reduce carbon emissions [2]. Zhang et al. tackled the low-carbon route optimization problem in cold chain logistics under Chinese traffic conditions, establishing a traffic-aware low-carbon route optimization model for cold chain logistics and designing an Improved Discrete Firefly Algorithm (IDFA) to optimize routes by integrating traffic conditions’ impacts on total distribution cost, product freshness, and carbon emissions [3]. Meng et al. addressed the green vehicle routing problem with time windows and customer preferences, aiming to solve the limitations of traditional algorithms in dynamic multi-objective optimization. They established a multi-objective mathematical model to minimize fuel consumption, carbon emissions, and costs, and designed a Q-learning-driven butterfly optimization algorithm (QLBOA) with dynamic Gaussian mutation and migration mechanisms [4]. However, with the continuous advancement of academic research and the gradual increase in social awareness, people are increasingly recognizing the limitations of focusing solely on the environmental and economic dimensions. The concept of sustainable development, which takes a more comprehensive, systematic, and long-term perspective, is gradually becoming the dominant direction in vehicle path planning and the transportation field as a whole. In 2001, Elkington proposed the “Triple Bottom Line" (TBL) for 21st-century enterprises: economic profit, environmental resources, and social impact, laying a theoretical foundation for building a greener, more efficient, and fairer transportation system. Many scholars have explored the “sustainable vehicle routing problem" (SUVRP). For instance, Yu et al. proposed an automated vehicle logistics system that integrates autonomous driving, logistics, and renewable energy for smart city services. This system optimizes routes by considering economic, environmental, and social objectives [5]. Hassana et al. developed a multi-objective optimization model and designed a greedy algorithm to address the green vehicle routing problem, with an additional focus on the social dimension [6].

In a logistics system, drivers play a crucial role, and their job satisfaction has a direct impact on work efficiency and the effectiveness of the entire logistics system. Usually, drivers’ job satisfaction is measured by considering the variability or fairness of their workload. For example, Li et al. addressed this issue by optimizing vehicle load balancing [7], while Siping et al. focused on fairness and efficiency in emergency logistics by optimizing a resilience assessment method in addition to load distribution [8]. However, focusing solely on workload magnitude differences (vehicle load) is one-sided, as the temporal fairness of drivers’ work schedules requires investigation. Therefore, this study will investigate the time-dependent network simultaneous pickup delivery vehicle path problem from a sustainable perspective. The objective function, grounded in the TBL criteria, incorporates human factors such as driver satisfaction through a multidimensional fairness function. By optimizing vehicle pickup and delivery orders and departure times, this study aims to reduce comprehensive delivery costs, minimize driver workload variability, and enhance overall logistics efficiency and service levels. This approach not only addresses the economic and environmental challenges of cold chain logistics but also emphasizes social equity, ensuring that temperature-sensitive products are delivered under optimal conditions to meet consumer demands.

To achieve these goals, a novel mixed-integer linear programming (MILP) model is developed, representing traffic congestion as time-varying travel speeds to minimize total distribution costs. Additionally, a “Heuristic Crossover Brainstorm Optimization Algorithm" (HCBSO) with an adaptive selection function and heuristic crossover strategy is proposed to maintain solution diversity and facilitate convergence. A departure time optimization strategy is also devised to enhance distribution efficiency, reduce costs, and mitigate risks of time loss and product degradation. Comprehensive experiments will be conducted to evaluate the model and algorithms, aiming to provide a scientific basis and practical optimization schemes for real-world cold chain logistics operations.

The paper is organized as follows. Sect 2 reviews the related literature. Sect 3 details the problems considered in this study and their corresponding mathematical models. Sect 4 presents the heuristic cross-brainstorming algorithm. Sect 5 conducts algorithm experiments. Sect 6 provides the study’s conclusions and future work.

## 2 Literature review

In 1981, Beasley proposed the “Time-Dependent Vehicle Routing Problem" (TDVRP). The focus of this problem lies in exploring vehicle routing scenarios where the travel time changes based on the departure time. Malandraki and Daskin focused on analyzing speed variations due to traffic congestion across distinct time intervals, formulating a mixed-integer programming model to accurately characterize the TDVRP [9]. Ichoua et al. modeled the dynamic road network as a time-varying speed function and introduced the “First-In-First-Out" (FIFO) rule [10].

In research on time-dependent vehicle routing, scholars have predominantly used step functions to model the dynamics of vehicle travel speeds. They utilized the FIFO principle to develop methods for computing travel times in time-varying road networks. These methods are applied to various logistics scenarios, with optimization algorithms designed to solve the resulting problems. For instance, Gmira et al. addressed the TDVRP with time windows, developing a tabu search heuristic algorithm based on relevant models [11]. Ma et al. focused on the TDVRP in shared autonomous electric vehicle services, emphasizing departure time and speed optimization to reduce costs by incorporating travel distance, energy consumption, and time factors [12]. Luo et al. addressed the “time-dependent green vehicle routing problem" (TDGVRP) under traffic congestion, aiming to develop vehicle scheduling strategies to reduce carbon emissions. To find solutions, they formulated a set partitioning formulation (SPF) and developed a “branch-price-and-cut" (BPC) algorithm [13]. Franco et al. focused on the bi-objective time-dependent vehicle routing problem with delivery failure probabilities, aiming to minimize operational costs and delivery failure rates. To address this complex problem, they developed a mixed-integer linear programming model [14]. Wang et al. addressed the dynamic TDGVRP in waste collection, aiming to improve construction waste transportation efficiency and reduce carbon emissions. They formulated a 0-1 programming model and designed a two-stage solution algorithm [15]. Li et al. addressed the time-dependent vehicle routing problem with time windows and spatio-temporal distances, aiming to minimize travel time and maximize customer satisfaction, they developed relevant models and designed an improved multi-objective evolutionary algorithm (IMOEA) for solution [16]. Liu et al. targeted the TDGVRP and designed an effective adaptive large neighborhood search (ALNS) algorithm to reduce carbon emissions [17]. Said et al. corrected the errors in the computer implementation of the branch and price algorithm for the TDGVRP [18]. Castellucci et al. targeted the TDVRP, acknowledging the challenges posed by daily traffic congestion to urban logistics operators and developing an exact approach based on logic-based vendor decomposition and a branch-and-Benders-cut algorithm [19].

Recently, many scholars have applied the TBL criterion to vehicle path optimization problems. The Sustainable Vehicle Routing Problem (SUVRP) has been widely explored. For example, Ahteshamul Haq et al. addressed the optimization of India’s Sustainable Development Goals (SDGs) by proposing a neutrosophic programming (NP) model to handle uncertainties in SDG optimization, which aims to provide policymakers with a comprehensive and flexible decision-making tool by optimizing India’s 2030 socio-economic and environmental goals [20]. İlker et al. considered environmental and social sustainability, developed a hybrid linear programming model and proposing a large-scale case-solving method based on a clustering algorithm [21]. Tingting et al. addressed refrigerated pharmaceutical logistics distribution, devising a SAVNS-based hybrid heuristic algorithm to balance economic and environmental objectives [22]. Liu et al. addressed fresh product logistics and distribution, introducing a customer-society dimension via a prospect theory-based nonlinear function under the triple bottom line framework [23]. Dragan et al. proposed a multi-objective home healthcare path optimization model, considering both physician-patient satisfaction and sustainability, designing an improved adaptive non-dominated sorting genetic algorithm to solve the problem [24]. Pamučar et al. proposed a new model to determine vehicle routes for time-dependent urban logistics path optimization, which integrates the parameters of environment, health, space usage, and logistics operation cost [25]. Soysal et al. proposed a novel model for the one-to-one concurrent pickup-and-delivery problem, which considers variable speed, road classification, and environmental, economic, and social aspects through a mixed-integer programming framework [26]. Mohammad et al. considered environmental, economic, and social dimensions and designed an adaptive memory social engineering optimizer to address them [27]. Geonhwa et al. addressed the sustainable vehicle path problem under real-time road conditions and proposed a heuristic algorithm based on restrictive inheritance. In addition, a number of scholars have also applied the TBL criterion to reverse logistics path optimization problems [28]. Yu et al. developed a dual-objective mixed-integer linear programming model for integrated express delivery and packaging recycling optimization, designing a multi-objective hybrid-heuristic algorithm to solve it [29]. Mohd Arif Khan et al. addressed supply-demand uncertainty in multi-objective fractional transportation problems, aiming to develop a flexible model aligned with decision-makers’ preferences by considering objectives such as transportation cost, delivery time, deterioration, environmental impact, and social concerns [30].

In addition to economic, environmental, and social aspects, participant satisfaction is also crucial for system sustainability. Most studies related to this paper (TDVRP, SUVRP) focus only on customer satisfaction, with few addressing driver job satisfaction or fairness. In reality, driver work condition variability includes workload, working hours, and rest time. In time-dependent road networks, vehicle departure times impact drivers’ waiting times. Assuming all vehicles depart simultaneously leads to unnecessary waiting and exacerbates driver inequity, an area that needs further exploration.

## 3 Problem description

### 3.1 Description of the problem

**Fig 1:**
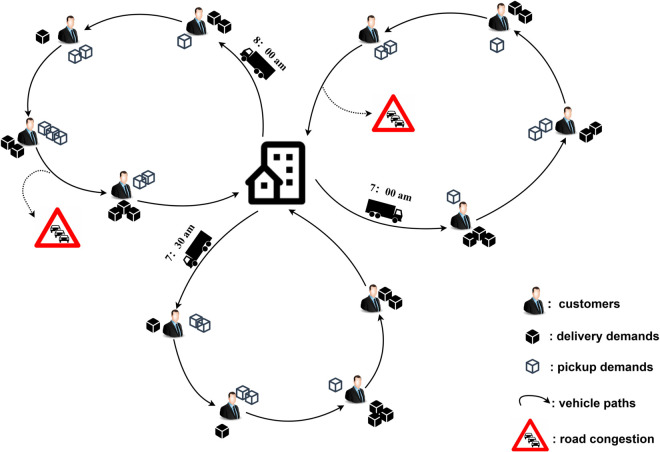
Vehicle distribution schematic.

The cold chain distribution center is equipped with numerous identical cold-chain logistics vehicles. Their task is to offer pickup and delivery services to a set of customers whose locations, delivery, and pickup requirements, along with service time windows, are known. Given that the traffic conditions in an urban road network are dynamic over time, the driving velocities of vehicles vary according to different time intervals. The goal is to minimize overall cost, including economic, environmental, social, and driver satisfaction factors. This is achieved by optimizing vehicle departure times and route sequences while ensuring compliance with vehicle load constraints and customer time window requirements. A schematic of the vehicle distribution process is shown in Fig 1.

The symbols and interpretations involved in the article are shown in Table 1.

**Table 1:** Symbols and interpretations.

Symbols	Interpretations
*N*	Set of customer points: N={1,2,…,n}
*K*	Set of vehicles: K={k∣k=1,2,…,K}
*T*	Set of times: $T=\{T_{0},T_{1},\ldots ,T_{h-1},T_{h}\}$
	Operating time window of distribution centers
$\left[a_{i},b_{i}\right]$	Time window for customer point *i*
*Q*	Vehicle’s maximum load capacity
*q*_*i*_	Demand at customer point *i*
*p*_*i*_	Pickup demand at customer point *i*
*s*_*i*_	Service time at customer point *i*
*d*_*ij*_	Distance from point *i* to point *j*
*Q*_*ijk*_	Load carried by vehicle *k* from point *i* to point *j*
*a*_*ik*_	Arrival time of vehicle *k* at point *i*
*b*_*ik*_	Service start time of vehicle *k* at point *i*
*wt*_*ik*_	Waiting time of vehicle *k* at customer point *i*
*lt*_*ik*_	Departure time of vehicle *k* from point *i*
*x*_*ijk*_	Binary variable: *x*_*ijk*_ = 1 if vehicle *k* travels from point *i* to point *j*, otherwise *x*_*ijk*_ = 0

### 3.2 Calculation of travel time

In the context of real-world traffic monitoring, congestion levels are periodically refreshed. This paper models a similar situation, assuming that vehicles move at varying speeds at different times. Following the “First-In-First-Out" principle, we adopt the dynamic road network vehicle travel time calculation approach by Ichoua. The operational time of the distribution hub is segmented into *H*intervals, each of length *Z*, denoted as $T=\left\{T_{0},T_{1},...,T_{H-1},T_{H}\right\}$, where $\left(T_{h-1},T_{h}\right)$ represents the h-th interval. *d*_*ij*_ is the distance between *i* and *j*, *T*_*ik*_ is the departure time of the vehicle *k* from point *i*, falling within $\left[T_{h-1},T_{h}\right]$, $L_{ij}^{h}$ is the remaining distance that vehicle *k* must cover after interval *h* to finish the entire road segment (i,j), $V_{ijk}^{h}$ is the speed of the vehicle *k* traveling from point *i* to *j*, during interval *h*, and $D_{ijk}^{h}$ is the distance covered by vehicle *k* going from *i* to *j* in the *h* time period, $t_{ijk}^{h}$ is the traveling time of vehicle *k* going from *i* to *j* in the *h* time period, and *t*_*ijk*_ is the total travel time after completing the segment (i,j). The calculation of *t*_*ijk*_ is detailed in the subsequent steps:

- Step 1: Calculate the travel time during the departure interval. $D_{ijk}^{h}=V_{ijk}^{h}\left(T_{h}-T_{ik}\right)$, if $D_{ijk}^{h}\geq d_{ij}$,tijk=dij/dijVijkhVijkh, output *t*_*ijk*_. Otherwise, $L_{ij}^{h}=d_{ij}-D_{ijk}^{h}$, $t_{ijk}^{h}=T_{h}-T_{ik}$, and proceed to Step 2.
- Step 2: Calculate the travel time for subsequent intervals.
  (1)Initialize λ=1;
  (2)$D_{ijk}^{h+\lambda }=V_{ijk}^{h+\lambda }Z$, if $D_{ijk}^{h+\lambda }\geq L_{ij}^{h+\lambda -1}$, then tijkh+λ=Lijh+λ−1/Lijh+λ−1Vijkh+λVijkh+λ, and $t_{ijk}=\sum_{h\in T}T_{ijk}^{h}$, output *t*_*ijk*_.
  Otherwise, $L_{ij}^{h+\lambda }=L_{ij}^{h+\lambda -1}-D_{ijk}^{h+\lambda }$, $t_{ijk}^{h+\lambda }=Z$, increment *λ* by 1, repeat step 2.

### 3.3 Calculation of economic cost

#### 3.3.1 Fixed cost.

The fixed cost, denoted as *C*_1_ pertains to elements such as the fundamental upkeep of delivery vehicles and the wages of drivers. It is associated with the quantity of vehicles utilized. The computational procedure is presented as follows:

$$C_{1}=C_{f}\sum_{k\in K}\sum_{j\in N}\sum_{h\in T}x_{0jk}^{h}$$ (1)

#### 3.3.2 Refrigeration cost.

Given that cold-chain products are perishable, highly susceptible to spoilage, and require strict temperature control throughout the distribution process, a specific amount of refrigeration expenses will be incurred. Refrigeration costs can be categorized into three components. The first component is the refrigeration cost during transit. The second is the refrigeration cost when the vehicle is in the waiting state. The third is the refrigeration cost resulting from the opening and closing of the compartment door during vehicle service operations. The following elaborates on the detailed calculation procedure for refrigeration costs:

$$C_{2}=C_{21}+C_{22}+C_{23}$$ (2)

$$C_{21}=P_{2}\sum_{k\in K}\sum_{i,j\in N}\sum_{h\in T}t_{ijk}^{h}x_{ijk}^{h}$$ (3)

$$C_{22}=P_{2}\sum_{k\in K}\sum_{i,j\in N}\sum_{h\in T}\max\left(\left(a_{j}-at_{jk}\right)x_{ijk}^{h},0\right)$$ (4)

$$C_{23}=P_{3}\sum_{i\in N}s_{i}$$ (5)

#### 3.3.3 Goods damage cost.

Cold chain products are highly sensitive to temperature. Once the temperature exceeds a certain level, there will be a certain degree of loss. We adopt the continuous exponential loss function $D(t)=D_{0}e^{-\varepsilon t}$, where D(t) represents the product quality at time *t*, *D*_0_ indicates the product quality upon exiting the distribution center, *t* represents the length of time the product is transported, and ε denotes the spoilage rate of the product, affected by environmental factors. The cost of goods damage during the entire distribution process is:

$$C_{3}=C_{31}+C_{32}$$ (6)

$$C_{31}=\sum_{k\in K}\sum_{h\in T}\sum_{i\in N}{Y^{h}}_{ik}P_{1}\left(q_{i}+p_{i}\right)\left(1-e^{-\varepsilon_{1}s_{i}}\right)$$ (7)

$$C_{32}=\sum_{k\in K}\sum_{h\in T}\sum_{i,j\in N}P_{1}Q_{ijk}\left(1-e^{-\varepsilon_{2}t_{ijk}}\right){x^{h}}_{ijk}$$ (8)

### 3.4 Calculation of environmental cost

In which the environmental cost mainly consists of the fuel quantity, which is calculated by the Comprehensive Emission Model, and the fuel consumption ${f\;}_{ijk}^{h}$ of vehicle *k* from *i* to *j* in the time period *h* can be expressed as:

$$C_{4}=C_{r}C_{p}e\sum_{i,j\in N}\sum_{k\in K}\sum_{h\in T}x_{ijk}^{h}\cdot {f\;}_{ijk}^{h}$$ (9)

$${f\;}_{ijk}^{h}=\omega \gamma \mu \left\{\left(a_{ij}+g\sin\theta_{ij}+gC_{r}\cos\theta \right)\left(m_{0}+Q_{ijk}\right)D_{ijk}^{h}+0.5C_{d}\rho A{\left(v_{ijk}^{h}\right)}^{2}D_{ijk}^{h}\right\}\;+\omega \gamma KNVD_{ijk}^{h}/v_{ijk}^{h}$$ (10)

### 3.5 Calculation of social cost

The social dimension is calculated as a measure of the cost of accident risk from *i* to *j*. The magnitude of the cost depends on the length of the travel distance and the load of vehicle *k*. Modeling social costs with reference to Hassana et al, where *ψ* is the proposed monetization factor of the risk per unit of accident, and *Q*_*ijk*_ denotes the load on vehicle *k* leave point *i* to point *j*.

$$C_{5}=\sum_{i\in N}\sum_{j\in N}\sum_{k\in K}\psi \cdot d_{ij}\cdot Q_{ijk}$$ (11)

### 3.6 Calculation of fairness cost

To mitigate the wastage of human resources and manpower stemming from extended driver waiting times during the work period and to address the issue of perceived unfairness arising from workload disparities, this research assesses the fairness of drivers’ work across three aspects: workload, total working duration, and the proportion of waiting time. The calculation of the latter is presented in Eq (12).

$$C_{6}=\sum_{k\in K}\left\{\begin{array}{c} \omega_{1}\left[Max(R_{k})-Min(R_{k})\right]+\\ \omega_{2}\left[Max(T_{k})-Min(T_{k})\right]+\\ \omega_{3}\left[Max(\frac{Z_{k}}{T_{k}})-Min(\frac{Z_{k}}{T_{k}})\right] \end{array}\right\}$$ (12)

#### 3.6.1 Mathematical model.

$$MinC=C_{1}+C_{2}+C_{3}+C_{4}+C_{5}+C_{6}$$ (13)

$$\sum_{k\in K}\sum_{j\in N}\sum_{h\in T}x_{jik}^{h}=1,\forall i\in N$$ (14)

$$\sum_{j\in N}\sum_{h\in T}x_{0jk}^{h}=1,\forall k\in K$$ (15)

$$\sum_{i\in N}\sum_{h\in T}x_{ijk}^{h}-\sum_{i\in N}\sum_{h\in T}x_{jik}^{h}=0,\forall k\in K,\forall j\in N$$ (16)

$$\sum_{i\in N}\sum_{h\in T}x_{i,n+1,k}^{h}=1,\forall k\in K$$ (17)

$$\sum_{j\in N}Q_{0jk}=\sum_{j\in N}\sum_{h\in T}q_{i}x_{0jk}^{h}\leq Q,\forall k\in K$$ (18)

$$\sum_{i\in N}Q_{i0k}=\sum_{i\in N}\sum_{h\in T}p_{i}x_{i0k}^{h}\leq Q,\forall k\in K$$ (19)

$$0\leq Q_{ijk}\leq Q,\forall i,j\in N,\forall k\in K$$ (20)

$$at_{ik}+wt_{ik}=bs_{ik},\forall i\in N,\forall k\in K$$ (21)

$$bs_{ik}+s_{i}=lt_{ik},\forall i\in N,\forall k\in K$$ (22)

$$E\leq a_{i}\leq bs_{ik}\leq b_{i}\leq L,\forall i\in N,\forall k\in K$$ (23)

$$x_{ijk}^{h}\in \left\{0,1\right\},\forall i\in N,\forall k\in K,\forall h\in T$$ (24)

Where Eq (13) functions as the objective function. The aim is to achieve the lowest possible total cost, which encompasses economic cost, environmental cost (cost of carbon emissions), social cost, and fairness cost; Eq (14) imposes a constraint that each customer should be visited exactly once; Eq (15), Eq (16), Eq (17) specify that each vehicle departs from 0 respectively; ensure flow balance at the node; eventually, each vehicle must arrive *n*  +  1; Eq (18) denotes the loading formula applicable when vehicle *k* departs from the distribution center with a load that is lower than the vehicle’s rated load; Eq (19) represents the loading formula for vehicle *k* when it returns to the distribution center with a load that is less than the vehicle’s rated load *Q*; Eq (20) represents the loading capacity constraint during the working period of the vehicle *k*; Eq (21) and Eq (22) indicates the continuity of the distribution time; and Eq 23 represents the formula of the vehicle *k* start service time to *i* must satisfy the time window $\left[a_{i},b_{i}\right]$ at that customer point and also satisfy the working time window [*E*,*L*] at the distribution center; Eq (24) denotes a 0-1 decision variable.

## 4 Algorithm design

The “cold chain time-dependent vehicle routing problem with simultaneous pickup and delivery and time windows" (CC-TDVRPSDPTW) is NP–hard problem. Exact algorithms and solvers, such as Gurobi, can handle small-scale versions of this problem. Nevertheless, for large-scale cases, it is challenging to determine an optimal solution within a practical time limit. Brainstorming algorithms belong to heuristic algorithms. They are simple, have strong evolutionary capabilities, and perform well in finding optimal solutions. However, they rely on the initial solution and can get trapped in local optima. To address these issues, this study proposes a heuristic cross-brainstorming optimization algorithm.

### 4.1 Initial solution generation

The quality of the initial solution affects both the final solution quality and algorithm efficiency. Randomly generating the initial population may produce many invalid individuals and increase convergence time. To improve feasibility, integer coding was used to construct the initial solution step-by-step, starting with departure times and node arrival times. This ensures all initial solutions are feasible. Moreover, randomness in the algorithm promotes population diversity and prevents it from being trapped in local optima.

- Step 1: Arbitrarily choose a vehicle. Then, arrange the customers in an ascending sequence based on the magnitude of the time window on the left-hand side of each customer. Next, randomly pick one of the first three customers from the sorted list to serve as the starting customer for the vehicle’s service, and include this customer in the vehicle’s initial service route.
- Step 2: Proceed through the remaining unvisited customers in sequential order. For each unvisited customer, compute the estimated arrival time when traveling from the current customer. Subsequently, verify whether this arrival time meets the customer’s time-window constraints and the vehicle’s load capacity limit. If both conditions are satisfied, include the customer in the current route and update the route-related information. Conversely, if either condition is not met, assign a new vehicle to serve that particular customer.
- Step 3: Repeat steps 1 and 2 and continue to arrange service paths for each vehicle until all customers are arranged into different paths, completing the initial path planning.

### 4.2 Fitness selection function

In order to increase the probability of intra-group discussion in the early stage of the algorithm and enhance its diversity. Increase the probability of discussion within the group in the later stage and achieve convergence in the later stage. The settings are as follows:

$$p\_one=\sigma +\frac{gen}{Maxgen}\times \lambda$$ (25)

### 4.3 Heuristic crossover strategy

In a brainstorming algorithm, crossover mimics biological mating to generate new individuals and enhance global search. However, a fixed search direction may lead to unproductive searches and local optima. To address this, this study proposes an enhanced search strategy that sets the initial search direction of offspring to bidirectional. This broadens the search scope, accelerates convergence, and improves performance.

Two paternal chromosomes, $parent1=\left\{x_{11},x_{12},x_{13},...x_{1N}\right\}$ and $parent2=\left\{x_{21},x_{22},x_{23},...x_{2N}\right\}$, were selected to produce zygotic chromosomes as follows:

- Step 1: Randomly select one gene of the parent *x*_*i*_ as the first gene of the offspring (*pos* = 1);
- Step 2: Find the location of *x*_*i*_ at *x*_1*i*_ and *x*_2*i*_ in the parent *parent*1 and *parent*2, respectively. When a gene is located at the terminal position of the parental individual, its subsequent position will be the initial position of the gene in the parent. Conversely, if a gene is in the first position in the parental individual, its previous position will be the terminal position of the gene in the parent. Follow the instructions below to complete the gene update at *pos* = 2 in the offspring:
  Update the offspring *child*1: Select the genes of *x*_1,*i* + 1_ and *x*_2,*i* + 1_ in the parent, calculate $dist\left(x_{i},x_{1,i+1}\right)$ and $dist\left(x_{i},x_{2,i+1}\right)$ respectively and compare the size, the smaller distance between *x*_1,*i* + 1_ and *x*_2,*i* + 1_ will be the next gene of *x*_*i*_ and update *child*1;
  Update the offspring *child*2: Select the genes of *x*_1,*i*−1_ and *x*_2,*i*−1_ in the parent, calculate $dist\left(x_{i},x_{1,i-1}\right)$ and $dist\left(x_{i},x_{2,i-1}\right)$ respectively and compare the size, take the smaller distance between *x*_1,*i*−1_ and *x*_2,*i*−1_ will be the next gene of *x*_*i*_ and update *child*2;
- Step 3: Remove *x*_*i*_ from the parents *parent*1 and *parent*2, update *parent*1 and *parent*2;
- Step 4: Use the genes obtained in step 2 as a new zygote starting point (*pos* = *pos* + 1);
- Step 5: Cycle steps 2-step 4 until the gene counts of *child*1 and *child*2 are *N*, and output the offspring path.

### 4.4 Departure time optimization strategy

In vehicle path research, most studies assume that all vehicles leave the distribution center simultaneously. However, departure time is crucial for avoiding traffic congestion and reducing costs. Optimizing departure times can significantly cut fuel consumption and travel time while meeting customer needs. Therefore, this paper proposes a departure-time optimization strategy to improve distribution efficiency and reduce costs through smart scheduling.

The vehicle departs from the distribution center to the first customer point to provide pickup and delivery services for them, according to the distribution of congestion time period and the time window requirement of the first customer point, the earliest departure time of the vehicle *ES* and the latest departure time *LS*, and the nearest congestion time period $\left[T_{1},T_{2},\right]$, and the optimal departure time *t*_*best*_ calculated as shown in Eq (28) according to the travel time formula:

$$t_{best}=\left\{ \begin{array}{c} ES,ES\leq T_{1}orES\geq T_{2}\\ LS,T_{1}<ES<LS<T_{2}\\ T_{2},ES<T_{2}<LS \end{array}\right.$$ (26)

The optimal departure time *t*_*best*_ is then evaluated to ensure that the vehicle satisfies the initial load constraints and time window constraints, and the evaluation function is shown in Eq (29) as follows:

$$\rho_{1}Load(t_{best})+\rho_{2}TW(t_{best})\leq 0$$ (27)

where $\rho_{1}$ and $\rho_{2}$ are the penalty coefficients for violating the load constraint and the time window constraint, *Load*(*t*_*best*_) and *TW*(*t*_*best*_) are the values for violating the load constraint and the time window constraint at departure time *t*_*best*_.

### 4.5 Algorithmic process

**Fig 2:**
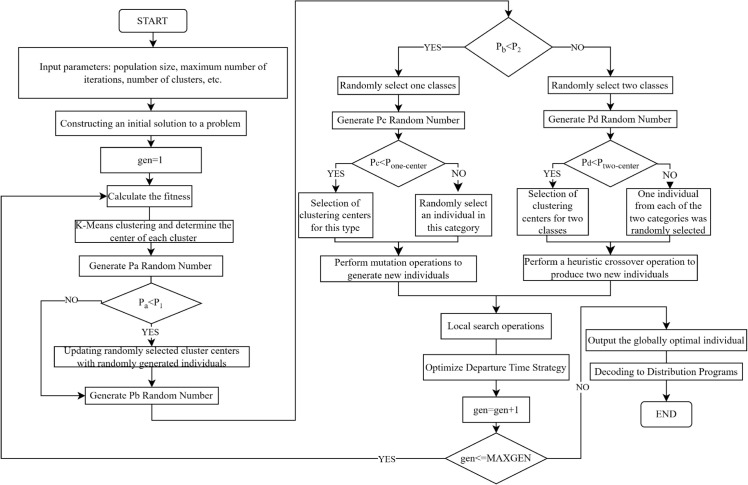
Algorithmic flow chart.

**Fig 3:**
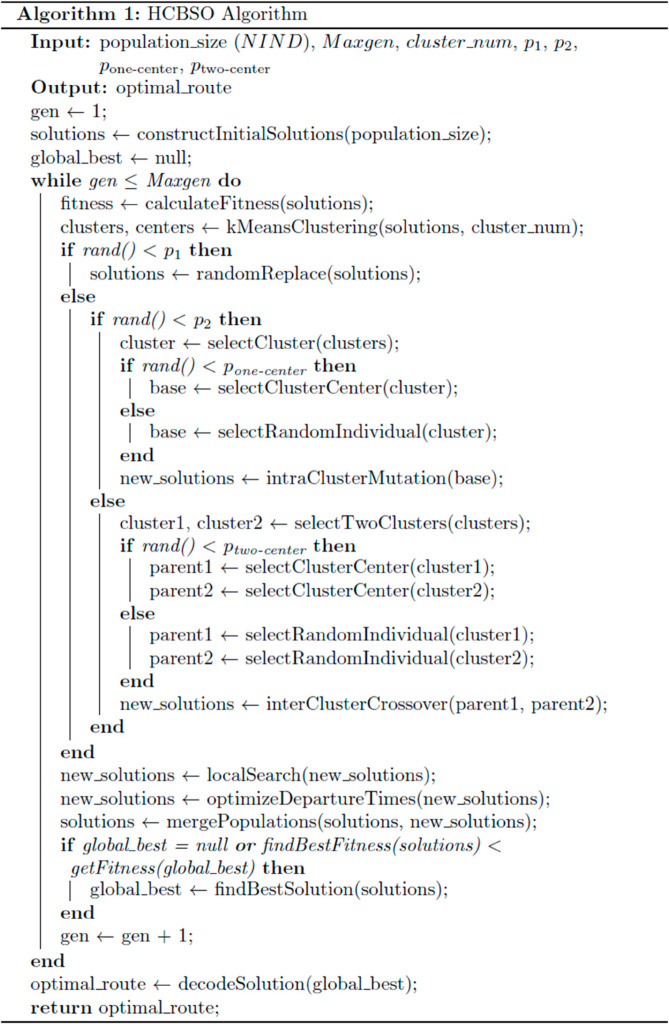
Algorithmic pseudocode.

The flow of the heuristic cross-brainstorming optimization algorithm is shown in Fig 2, and the pseudocode of the heuristic cross-brainstorming optimization algorithm is shown in Fig 3.

## 5 Experiments

### 5.1 Experiment setup

The program is implemented using MatlabR2023b programming, and Gurobi is implemented using Python3.10 programming, both run under Intel(R) Core (TM) i7-14700HX 2.10 GHz (16.0 GB), Windows 11 operating environment.

Model parameters: fairness parameter *ω*_1_ = *ω*_2_ = *ω*_3_ = 1 / 3, unit accident risk monetization coefficient ψ=0.0005, each vehicle cost is 200 yuan. The refrigeration cost for unit transportation and waiting time is 10 yuan per hour, and the refrigeration cost for unit service time is 15 yuan per hour. The unit price of cold-chain products is 20 yuan. The spoilage rate of products during transportation is 0.1%, and the spoilage rate of products during the process of loading and unloading is 0.2%, unit fuel consumption cost $C_{r}=6.43yuan\text{/L}$, unit carbon emission cost *C*_*p*_ = 0.05*yuan*/*kg*, carbon emission coefficient *e* = 3.9059*CO*_2_/*kg*. The meanings and values of vehicle fuel-related parameters are detailed in the S1 Appendix.

Algorithm parameters: the maximum number of iterations *Maxgen* = 100−300, the number of populations *NIND* = 50, the number of clusters cluster_num=5, the random replacement probability p_re=0.1, the inter-group discussion probability p_two=1 − p_one, the cluster center probability p_one_center=0.8 in selecting one cluster, the cluster center probability p_two_center=0.2 in selecting two clusters, the probability of the most intra-group discussion σ=0.1, the maximum intra-group discussion probability λ=0.8.

### 5.2 Validity experiment

To confirm the effectiveness of the model and algorithm presented in this paper, a comparison was made between the solution results of the algorithm proposed herein and Gurobi’s exact algorithm. This comparison was conducted on the TDVRPTW case, with the objective of minimizing the total distance traversed by the vehicle. Since there is no standard test case for TDVRPTW, this paper constructs the TDVRPTW case based on the C, R, and RC series of test data from the Solomon dataset, which can be downloaded from https://neo.lcc.uma.es/vrp/. The data changes are as follows: add the time-varying speed to the original data, set the working time of the distribution center [0,l], and divide it into three time periods: [0,l/l33), [l/l33,2l/2l33), [2l/2l33,l], and three vehicle speeds: $v_{1}=25$, $v_{2}=10$, $v_{3}=36$.

Configure the Gurobi solver such that its maximum running time is set to 3600 seconds. In the event that this time limit is exceeded, the solver will output the current optimal solution, and the results are detailed in Table 2, where N denotes the number of customers, Obj, K, t/s denotes the objective function, vehicles number used, the running time of the code, respectively, and GAP denotes the deviation of the optimal solution.

**Table 2:** Comparison with Gurobi’s exact algorithm.

		Gurobi’s			HCBSO			
Example	N	Obj	K	t/s	Obj	K	t/s	GAP
C105	5	42.42	1	<1	42.42	1	<1	0.00%
	10	58.33	1	1.72	58.33	1	2.74	0.00%
	15	142.14	2	6.62	142.14	2	4.32	0.00%
	25	191.81	3	47.53	191.81	3	9.08	0.00%
C201	5	70.45	1	<1	70.45	1	<1	0.00%
	10	152.29	2	1.10	152.29	2	3.07	0.00%
	15	189.27	2	4.72	189.27	2	4.18	0.00%
	25	215.54	2	35.12	215.54	2	9.67	0.00%
R105	5	141.85	2	<1	141.85	2	<1	0.00%
	10	231.53	2	4.12	231.53	2	3.10	0.00%
	15	321.98	3	77.86	319.23	3	4.36	0.86%
	25	491.56	3	3600+	464.67	3	8.84	5.47%
R201	5	141.85	2	<1	141.85	2	<1	0.00%
	10	249.20	2	1.94	249.20	2	2.72	0.00%
	15	326.46	3	64.44	317.81	3	4.22	2.65%
	25	483.12	4	3600+	463.48	4	8.72	4.07%
RC201	5	89.13	1	<1	89.13	1	<1	0.00%
	10	183.14	2	3.03	183.14	2	2.92	0.00%
	15	220.67	2	6.52	220.67	2	4.22	0.00%
	25	369.30	3	3600+	359.23	3	8.74	2.73%
RC207	5	89.13	1	<1	89.13	1	<1	0.00%
	10	144.67	1	3.23	144.67	1	3.20	0.00%
	15	201.34	2	3600+	189.06	2	4.23	6.10%
	25	313.98	3	3600+	305.47	3	9.19	2.71%
Avg								1.02%

From Table 2, we can see that for small problems, the Gurobi solver quickly finds the best solution. But for larger problems (more than 15 customers), Gurobi takes a long time and doesn’t always find a good solution within 3600 seconds. The HCBSO algorithm, on average, gives solutions that are 1.02% better than Gurobi’s, especially for R-type and RC-type data where it can be up to 6.10% better. This is because the customers in R-type and RC-type data are spread out, and HCBSO can plan routes better, avoiding traffic and reducing distances. The results show that the mathematical model and HCBSO algorithm used in this study are very effective for solving the TDVRPTW problem.

### 5.3 Algorithms comparison

To evaluate the superiority of the HCBSO algorithm, it was compared with the basic BSO algorithm and the Genetic Algorithm. The lc105 data from the PDPTW examples were selected, with the goal is total cost. For the BSO algorithm, the initial solution was randomly generated. The heuristic crossover strategy was deactivated and replaced with the Order Crossover (OX) strategy. Additionally, the departure-time optimization strategy was turned off. The parameters were set according to those of the HCBSO algorithm. For the GA algorithm, the initial solution was also randomly generated, with a mutation probability of 0.05 and a crossover probability of 0.9. Finally, a comprehensive comparison of the optimization outcomes of the three algorithms was conducted, and the results are presented in Table 3. Data download link: https://www.sintef.no/projectweb/top/pdptw/li-lim-benchmark/.

**Table 3:** Comparison of algorithms.

Algorithm	N	Vehicle	Vio_route	Vio_customer	Cost	Time	Distance
GA	15	8	0	0	2156.31	2025.1	430.67
BSO	15	2	0	0	996.52	1627.5	172.50
HCBSO	15	2	0	0	989.01	692.34	164.66
GA	30	13	2	5	35734.58	751.41	735.25
BSO	30	4	0	0	1847.11	1920.9	335.41
HCBSO	30	4	0	0	1702.83	705.60	326.66
GA	45	18	2	6	31856.80	6980.9	1347.95
BSO	45	6	0	0	2752.17	3800.1	703.09
HCBSO	45	6	0	0	2426.96	790.81	607.67

The results in Table 3 show that: The HCBSO algorithm demonstrates better optimization capabilities than the BSO algorithm and the GA algorithm. Specifically, compared with the other two algorithms, the HCBSO algorithm has smaller values in terms of the driving mileage (Distance) of vehicle routes, the required time (Time), and the total cost (Cost). Additionally, the number of route violations (Vio_route) and customer violations (Vio_customer) are also fewer. Therefore, this algorithm exhibits a more significant optimization effect in reducing energy consumption and costs during cold-chain logistics distribution operations.

**Fig 4:**
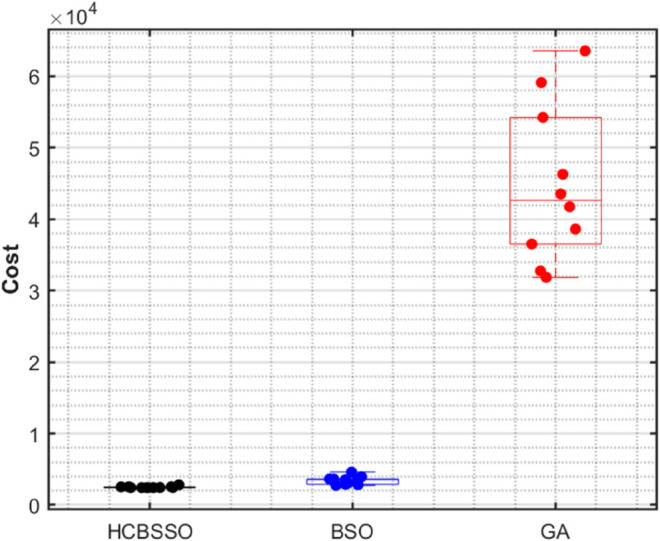
Statistical distribution of costs for different algorithms.

To evaluate the cost performance differences among the three algorithms, a non-parametric Kruskal–Wallis test was conducted on the lc105 instance (45 customers), with each algorithm executed 10 times. The null hypothesis is first proposed: *H*_0_: There is no significant difference in transportation costs among the HCBSO, BSO, and GA algorithms. Fig 4 illustrates the statistical distribution of transportation costs for the HCBSO, BSO, and GA algorithms, visually highlighting the overall advantage and stability of HCBSO in cost control.

The test revealed a statistically significant difference in transportation costs among HCBSO, BSO, and GA ($\chi^{2}$(2) = 19.91, p < 0.001), and the results are presented in Table 4. Dunn–Sidak post hoc analysis confirmed that HCBSO significantly outperformed BSO (p = 0.0379, CI = [–19.20, –0.40]) and GA (p < 0.0001, CI = [–29.30, –10.50]), while BSO also showed a significant advantage over GA (p = 0.0306, CI = [–19.50, –0.70]). These results demonstrate that HCBSO achieves superior and more stable cost optimization under cold-chain-specific constraints such as temperature control and time windows, making it a robust and effective solution for minimizing distribution costs in cold chain logistics.

**Table 4:** Dunn-sidak pairwise comparison of algorithm costs.

Algorithm Comparison Group	Mean Rank Difference	Confidence Interval	p	Result
HCBSO vs BSO	–9.80	[–19.20, –0.40]	0.0379	*H*_0_ is rejected
HCBSO vs GA	–19.90	[–29.30, –10.50]	0.0000	*H*_0_ is rejected
BSO vs GA	–10.10	[–19.50, –0.70]	0.0306	*H*_0_ is rejected

### 5.4 Sensitivity analysis

To further demonstrate the effectiveness of the model proposed in this paper for pickup and delivery problems, this chapter uses multiple sets of different types of Li & Lim PDPTW instances for verification (including lc type, lr type, and lrc type). Data download link: https://www.sintef.no/projectweb/top/pdptw/li-lim-benchmark/.

In order to verify the effectiveness of the vehicle departure strategy at different moments, under the premise of ensuring that other parameters remain unchanged, the strategy of this paper is compared with the vehicle departure strategy at a uniform start time (moment 0), and the results are detailed in Table 5, where N denotes the number of customers, Vehicle denotes the number of vehicles in use, Sta denotes the vehicle departure mode at a uniform moment (moment 0), Dif denotes the departure mode at different moments, IR denotes the improvement ratio.

**Table 5:** Comparative analysis of different modes of travel.

Example	*N*	Obj			Time			Vehicle	
		Dif	Sta	IR_Obj	Dif	Sta	IR_Time	Dif	Sta
lc101	50	2776.31	2777.36	0.04%	733.30	4792.26	84.70%	5	5
lc102	50	2722.18	2928.15	7.03%	1083.26	5089.55	78.72%	6	6
lc103	50	2654.30	2812.41	5.62%	466.29	5142.30	90.93%	6	6
lc201	50	3303.36	3418.29	3.36%	890.15	12093.57	92.64%	6	6
lc202	50	3144.54	3314.98	5.14%	588.01	12636.76	95.35%	6	6
lc203	50	3109.27	3268.91	4.88%	976.11	12470.45	92.17%	6	7
AVG				4.35%			89.08%		

The results in Table 5 show that: (1) In terms of the total cost (Obj), the strategy of departing at different times demonstrates significant advantages compared to the unified departure strategy, with an average improvement of 4.35% and a maximum improvement of 7.03%. (2) Regarding the total usage time of vehicles (Time), the strategy of departing at different times is also superior to the unified departure strategy, with an average improvement of 89.08% and a maximum improvement of 95.35%. (3) In terms of the number of vehicles used (Vehicle), the strategy of departing at different times is likewise better than the unified departure strategy. Therefore, implementing the strategy of vehicles departing at different times can effectively optimize logistics distribution routes, reduce the number of vehicles used, and shorten the working hours of carriers.

To validate the impact of the distribution of congestion time and the degree of congestion on path optimization, experimental control groups are set up: experimental group A: congestion distribution remains unchanged and congestion is aggravated (time distribution: [0,l/l33),[l/l33,2l/2l33),[2l/2l33,l], speed distribution: $v_{1}=25$,$v_{2}=5$,$v_{3}=36$). Experimental group B: congestion remains unchanged and congestion time is lengthened (time distribution: [0,l/l44),[l/l44,l/l22),[l/l22,3l/3l44),[3l/3l44,l], speed distribution: $v_{1}=25$,$v_{2}=10$,$v_{3}=10$,$v_{4}=36$).

The results in Table 6 show that compared to the benchmark group, Experimental Group A (with fixed congestion distribution but increased congestion levels) has significant increases in distribution cost and total travel time. The average increase in distribution cost is 7.04% (max 13.97%), and the average increase in travel time is 16.38% (max 30.09%). Experimental Group B (with prolonged congestion time but constant congestion levels) also shows notable increases. The average distribution cost rises by 5.68% (max 10.31%), and the average travel time increases by 11.75% (max 22.55%).

**Table 6:** Comparative analysis of different congestion conditions.

Instance	N	Experimental Group A				Experimental Group B				Benchmark Experimental	
		Obj	Time	IR_Obj	IR_Time	Obj	Time	IR_Obj	IR_Time	Obj	Time
lrc205	50	2774.92	3605.96	9.55%	30.09%	2644.13	2929.09	5.08%	13.93%	2509.93	2521.08
lrc206	50	2588.08	2489.51	13.97%	9.00%	2432.34	2417.98	8.46%	6.31%	2226.51	2265.47
lrc207	50	2599.29	2791.53	7.74%	15.40%	2470.09	3049.00	2.91%	22.55%	2398.12	2361.51
lrc208	50	1944.72	1940.74	6.55%	24.84%	2026.27	1835.38	10.31%	20.52%	1817.40	1458.72
lrc101	50	2263.77	761.86	3.19%	8.16%	2268.18	724.06	3.38%	3.37%	2191.52	699.66
lrc102	50	2358.16	978.30	1.25%	10.80%	2423.57	907.59	3.92%	3.85%	2328.58	872.68
AVG				7.04%	16.38%			5.68%	11.75%		

Therefore, traffic conditions have a significant impact on vehicle distribution efficiency. The intensification of traffic congestion and the extension of its duration will both lead to a substantial increase in the total travel time, which in turn will increase the distribution cost. It can be seen from this that avoiding traffic congestion is crucial for reducing the working hours of carriers and improving the efficiency of logistics distribution.

## 6 Conclusions and future work

This study addresses the sustainable vehicle routing problem in urban cold chain logistics by formulating a Cold Chain Time-Dependent Vehicle Routing Problem with Simultaneous Pickup and Delivery and Time Windows (CC-TDVRPSDPTW). A comprehensive Mixed-Integer Linear Programming (MILP) model is developed, integrating real-world complexities such as time-varying traffic conditions, simultaneous pickup and delivery operations, vehicle capacity constraints, and customer time windows. Additionally, the model incorporates sustainability metrics grounded in Triple Bottom Line (TBL) principles and integrates driver equity through a multidimensional fairness function. The modeling results demonstrate that accounting for time-dependent travel speeds significantly enhances delivery accuracy and reduces the risk of violating temperature-sensitive constraints.

To efficiently solve the model, a Heuristic Crossover Brainstorm Optimization (HCBSO) algorithm is proposed. Experimental results reveal that HCBSO not only achieves competitive solution quality compared to the Gurobi solver but also exhibits superior computational efficiency, particularly in large-scale instances, where exact methods face computational and memory constraints. Compared with classical Genetic Algorithms (GA) and traditional Brainstorm Optimization (BSO), HCBSO demonstrates enhanced global search capability and robustness, primarily due to its multi-stage initialization strategy and tailored heuristic crossover operator. Sensitivity analysis further indicates that staggered vehicle departure times under varying traffic scenarios effectively mitigate congestion, reduce delivery time, and lower total logistics costs. The analysis also shows that increasing congestion severity and duration leads to substantial cost escalation, underscoring the importance of departure time optimization in cold chain logistics.

The proposed model and algorithm serve as a valuable decision-support tool for logistics planners aiming to balance economic efficiency, environmental sustainability, and social responsibility. For practitioners, adopting the HCBSO algorithm enables better route planning and traffic avoidance strategies under dynamic urban conditions. Moreover, the integration of fairness and sustainability dimensions facilitates more equitable and sustainable logistics operations. The study’s findings can support the implementation of intelligent scheduling systems that dynamically adjust departure times and routes in response to real-time urban traffic dynamics. Policymakers may also leverage these results to design incentive mechanisms that encourage sustainable practices in the cold chain logistics sector.

Despite the promising results, this study has several limitations. The proposed model assumes deterministic time-varying traffic conditions, whereas actual urban logistics systems are often subject to stochastic uncertainties such as weather changes, road disruptions, and accidents, which may affect routing decisions and delivery times. Additionally, the HCBSO algorithm relies on fixed parameter settings, which may not be robust across diverse problem instances or logistics environments. Future research should aim to incorporate real-time dynamic elements—such as weather data, traffic incidents, and road network disruptions—to improve model adaptability and prediction accuracy. Furthermore, integrating HCBSO with machine learning techniques for intelligent parameter tuning could enhance its performance and robustness across varied applications. Extending the framework to accommodate multi-depot, multi-echelon, or multi-temperature logistics systems would also enhance its practical applicability. Moreover, the adoption of multi-objective optimization frameworks that balance cost, emissions, equity, and customer satisfaction can better capture trade-offs in sustainable logistics planning. These directions will contribute to the development of a more resilient, efficient, and intelligent cold chain logistics system under uncertainty.

## Supporting information

S1 Appendix Vehicle fuel-related parameter. (DOCX)
