# Supplementary material for: Research on the optimization of cold chain logistics distribution routes considering time-dependent networks and simultaneous pick-up and delivery from the perspective of sustainability
Source: PLoS One. 2025 Sep 5;20(9):e0330535. doi: 10.1371/journal.pone.0330535 (PMC12413086; doi:10.1371/journal.pone.0330535)
Supplement: S1 Appendix — (DOCX) [file pone.0330535.s001.docx]

| **Parameter** | **Interpretation** | **Values** |
| --- | --- | --- |
| $\omega$ | Heat released per gram of fuel burned | 6.43 |
| $\gamma$ | Conversion factor | 0.00135 |
| $\mu$ | $\mu=1/1000\eta\times\eta_{tf}$ | 0.00277 |
| $K$ | Engine friction coefficient | 0.2 |
| $N$ | Engine speeds | 33 |
| $V$ | Engine displacement | 5 |
| $A$ | Vehicle surface area | 3.912 |
| $C_{r}$ | Rolling resistance coefficient | 0.01 |
| $C_{d}$ | Drag coefficient | 0.7 |
| $m_{0}$ | Vehicle deadweight | 3500 |
| $a_{ij}$ | Accelerations | 0 |
| $\rho$ | Air density | 1.0241 |
| $g$ | Gravity constant | 9.81 |
| $\theta$ | Road slope | 0 |
